# Supplementary material for: Technically Challenging Percutaneous Interventions of Chronic Total Occlusions Are Associated with Enhanced Platelet Activation
Source: J Clin Med. 2023 Oct 29;12(21):6829. doi: 10.3390/jcm12216829 (PMC10648871; doi:10.3390/jcm12216829)
Supplement: Supplementary file 1 [file jcm-12-06829-s001.zip › Supplementary Tables S2 and S3.pdf]

Technically challenging percutaneous interventions of chronic total occlusions are associated with enhanced platelet activation

Supplementary Table S1. See .xls file named Supplementary Table S1 – Primers.xls

	J-CTO score 0 and 1<br>n = 24	J-CTO score 2 and 3<br>n = 26	p value
Age (years)	58.67 ± 8.07	64.65 ± 8.43	**0.019**
Male, n (%)	15 (62.5)	15 (57,7)	0.729
Hypertension, n (%)	21 (87.5)	21 (80.8)	0.516
Hyperlipidemia, n (%)	22 (91.7)	26 (100)	0.133
Obesity, n (%)	20 (83.3)	25 (96.2)	0.131
AMI in anamnesis, n (%)	10 (41.7)	12 (46.2)	0.749
DM in anamnesis, n (%)	9 (37.5)	10 (38.5)	0.944
ACEi / ARB, n (%)	21 (87.5)	23 (88.5)	0.917
BB, n (%)	19 (79.2)	26 (100)	**0.014**
Nitrates, n (%)	7 (29.2)	14 (53.8)	0.077
Statin, n (%)	18 (75)	25 (96.2)	**0.031**
Total number of stents	40	46	0.726
Total stent length (mm)	1 178	1 415	0.456
Average number of stents / PCI	1,67	1,77	
Average total stent length / PCI	49.08 mm	54.52 mm	

Supplementary Table S2. Demographic, clinical and lesion characteristics of recruited patients in the aspect of J-CTO score division. Data are displayed as the mean ± standard deviation (SD). Mann-Whitney test, Chi^2-test or Fischer's exact test were used as appropriate. AMI: acute myocardial infarction, DM: diabetes mellitus, J-CTO: Japanese chronic total occlusion score, ACEi: angiotensin-converting enzyme inhibitor, ARB: angiotensin receptor blocker, BB: beta-blocker, PCI: percutaneous coronary intervention.

	shorter procedure<br>n = 24	longer procedure<br>n = 20	p value
Age (years)	61.04 ± 9.79	63.20 ± 7.99	0.516
Male, n (%)	11 (45.8)	14 (70)	0.107
Hypertension, n (%)	20 (83.3)	16 (80)	0.775

Hyperlipidemia, n (%)	22 (91.7)	20 (100)	0.186
Obesity, n (%)	20 (83.3)	19 (95)	0.225
AMI in anamnesis, n (%)	9 (37.5)	10 (50)	0.405
DM in anamnesis, n (%)	6 (25)	9 (45)	0.163
ACEi / ARB, n (%)	20 (83.3)	18 (90)	0.521
BB, n (%)	22 (91.7)	18 (90)	0.848
Nitrates, n (%)	13 (54.2)	5 (25)	0.050
Statin, n (%)	18 (75)	19 (95)	0.071

Supplementary Table S3. Demographic and clinical characteristics of recruited patients in the aspect of the procedure duration. Shorter duration of intervention means 40 to 117 minutes, while procedure was considered long, if the duration was between 118 and 255 minutes. In case of 6 patients the duration of intervention is missing. Data are displayed as the mean ± standard deviation (SD). Mann-Whitney test, Chi^2-test or Fischer's exact test were used as appropriate. AMI: acute myocardial infarction, DM: diabetes mellitus, J-CTO: Japanese chronic total occlusion score, ACEi: angiotensin-converting enzyme inhibitor, ARB: angiotensin receptor blocker, BB: beta-blocker
